# Auditory steady-state response to chirp-modulated tones: A pilot study in patients with disorders of consciousness

**DOI:** 10.1016/j.nicl.2020.102261

**Published:** 2020-04-22

**Authors:** Marek Binder, Urszula Górska, Evaldas Pipinis, Aleksandras Voicikas, Inga Griskova-Bulanova

**Affiliations:** a Institute of Psychology, Jagiellonian University, ul. Ingardena 6, 30-060 Krakow, Poland; b Department of Neurobiology and Biophysics, Vilnius University, Sauletekio ave 7, LT-10257 Vilnius, Lithuania

**Keywords:** Auditory steady-state response, Chirp-modulated tones, Disorders of consciousness, Crs-r score, Auditory system, Electroencephalography

## Abstract

**Objective:**

Due to the problems with behavioral diagnosis of patients with prolonged DOC (disorders of consciousness), complementary approaches based on objective measurement of neural function are necessary. In this pilot study, we assessed the sensitivity of auditory chirp-evoked responses to the state of patients with severe brain injury as measured with CRS-R (Coma Recovery Scale - Revised).

**Methods:**

A convenience sample of fifteen DOC patients was included in the study. Auditory stimuli, chirp-modulated at 1–120 Hz were used to evoke auditory steady-state response (ASSR). Phase-locking index (PLI) estimates within low gamma and high gamma windows were evaluated.

**Results:**

The PLI estimates within a narrow low gamma 38–42 Hz window positively correlated with the CRS-R total score and with the scores of the Auditory and Visual Function subscales. In the same low gamma window, significant difference in the PLIs was found between minimally conscious (MCS) and vegetative state (VS) patients. We did not observe any between-group differences nor any significant correlations with CRS-R scores in the high gamma window (80–110 Hz).

**Conclusions:**

Our results support the notion that the activity around 40 Hz may serve as a possible marker of the integrity of thalamocortical networks in prolonged DOC patients.

**Significance:**

Auditory steady-state responses at gamma-band frequencies highlight the role of upper parts of auditory system in evaluation of the level of consciousness in DOC patients.

## Highlights

• Chirp-evoked responses were evaluated in patients with disorders of consciousness.
• PLI estimates in 38–42 Hz window positively correlated with the CRS-R total score.
• Gamma-range evoked activity may indicate the integrity of thalamocortical networks.

## 1 Introduction

Prolonged disorders of consciousness (DOC) is a group of neurological syndromes resulting from a severe brain damage (Giacino et al. 2014). Etiology varies within this group and may include both extensive cortical and subcortical injuries as well as focal brainstem lesions (Owen 2013). Two main forms of those disorders are distinguished: the vegetative state (VS; also labeled unresponsive wakefulness syndrome, UWS (Laureys et al. 2010)) indicates a condition in which patients retain wakefulness, yet they do not reveal any external signs of conscious awareness; the minimally conscious state (MCS) can be diagnosed when patients remain awake and may manifest behavior such as visual pursuit, or a reproducible response to command, suggesting the presence of conscious awareness (Gosseries et al. 2011). Nevertheless, discrimination between those two states is difficult due to sparsity and ambiguity of behavioral responses (Schnakers et al. 2009).

Recently, analysis of brain activity has become an intensely studied opportunity for the assessment of the state of DOC patients. Both neuroimaging, such as fMRI (Owen 2013), as well as electrophysiological (Laureys & Schiff 2012) techniques are used to explore variations in neural responses associated with various forms of DOC. It has been shown that the auditory system remains relatively robust to the nervous system lesions (Kotchoubey et al. 2005), thus methods addressing its responsivity in severe brain damage provide a valuable option for DOC patient studies. Among protocols involving auditory stimulation and concurrent evaluation of brain activity, auditory steady-state responses (ASSR) - an EEG-based measure – seems to represent a promising alternative. Contrary to the methods based on evaluation of transient electrophysiological responses, ASSRs require a relatively short duration of the recording session, and can be obtained with a relatively high signal-to-noise ratio. Numerous studies showed the sensitivity of ASSR protocols to the altered states of brain functioning, both of physiological origin like sleep (Cohen et al. 1991, Picton et al. 2003b, Binder & Górska 2018), brain injury (Firsching, 1989, Harada et al. 1994, Serafini et al. 1994), schizophrenia (O'Donnell et al. 2013, Griskova-Bulanova et al. 2018) or being a result of pharmacological manipulations (such as anesthesia; Plourde 2006; Plourde et al. 2008a). Our recent study demonstrated that 40 Hz ASSR is sensitive to the state of DOC patient as measured with CRS-R total score: higher results in CRS-R and its subscales were associated to the higher phase-locking indices (PLI) of ASSR response (Binder et al. 2017).

This result, while showing a promising prospect of using gamma-range stimulation in DOC patients, provides quite a narrow window (40 Hz) to investigate the responsivity of the damaged human brain. The stimulation protocols based on chirp-modulation of the tones (Artieda et al. 2004), on the contrary, allow testing a wide range of stimulation frequencies simultaneously. The chirp-evoked responses have been tested in humans (Alegre et al. 2008) and rats (Pérez-Alcázar et al. 2008) allowing to capture physiologically relevant activity not only in the low gamma, but also the high gamma ranges, thus providing a more global insight on the brain responsivity. An important advantage of this approach is its efficacy: a wide range of stimulation rates is tested during the time period comparable to that of standard 40 Hz ASSR testing. This is essential in case of DOC patients whose activation levels are prone to decrease during EEG session.

In this pilot study, we assessed the sensitivity of chirp-evoked wide range (1–120 Hz) response to the state of patients with severe brain injury as measured with CRS-R. We specifically focused on the low gamma and high gamma ranges and we expected 1) that in concordance with the previous study, the response in the low gamma range (30–50 Hz) would show a correlation with the state of the patient, and 2) the response would differentiate between vegetative and minimally conscious patients. We expected the high gamma range (80–110 Hz) response to be similar in both patient groups. As previous reports suggest (Artieda et al. 2004, Luke et al. 2017), this response appears to be mainly dependent on brainstem sources whose integrity is rarely affected by the brain injuries resulting in DOC (Guérit et al. 1993, Kotchoubey et al. 2005, Rossi et al. 2015, André-Obadia et al. 2018).

## 2 Methods

### 2.1 Subjects and crs-r assessment

The convenience sample of prolonged DOC patients consisted of 15 subjects (4 females). Mean age was 45.53, SD: 15.45. Detailed information about the patients, including the sex, age, etiology, diagnosis, CRS-R results, time after the injury, and the interval between EEG testing and CRS-R administration was provided in Binder et al. (2017). For each patient an informed consent was acquired from their legal surrogates. The study design was approved by the local review board at the Institute of Psychology, Jagiellonian University and complied with the provisions of the Declaration of Helsinki. Before commencing the experiment each candidate patient underwent otoacoustic emissions audiological testing with OtoRead™ (Interacoustics, Middelfart, DK). The testing procedure involved proprietary TEOAE protocol (Kemp 1978). The threshold for “Pass” result (implying intact outer hair cells) was SNR of 4 dB at any three out of the six frequencies (range 0.5–4 kHz) used. “Pass” result was the inclusion criterion for the study.

The state of the patients was determined using the Polish version of the Coma Recovery Scale-Revised (Binder et al., 2018) within one week on average relative to the date of EEG measurement. CRS-R provides differential diagnosis, prognostic assessment and treatment planning in patients with disorders of consciousness (Giacino and Kalmar 2006). The scale contains six subscales that allow for an assessment of auditory, visual, motor, oromotor/verbal, communication and arousal functions. CRS-R consists of 23 items; the lowest-scored ones represent reflexive responses while the highest-scored behaviors involving cognitive processing (Giacino et al. 2004).

### 2.2 Stimulation

**Fig. 1:**
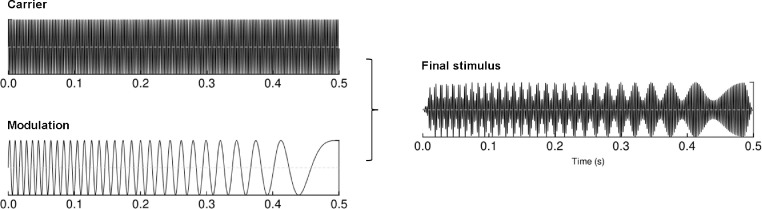
The structure of an amplitude-modulated chirp stimulus used in the study.

The auditory stimuli were designed in the MATLAB environment (The MathWorks, Inc.). Each individual stimulus consisted of 440 Hz tone amplitude modulated with a linear chirp that decreased in frequency from 120 to 1 Hz (see Fig. 1). Stimulus duration was 500 ms, with 15 ms onset/offset linear ramps to avoid clicks. Each patient was presented with 300 stimulus repetitions with 700–1000 ms variable inter-stimulus intervals (in 100 ms steps). Patients were seated on a wheelchair or in their bed in the upright position. Recording took place in a separate room or in the patient room, with only the patient and two experimenters present. Ambient noise levels were not monitored at either recording session. EEG acquisition was performed when patients had eyes open to ensure they were in the awake state. Experimenters were blind to the results of the CRS-R scores at the time of recording and data processing.

### 2.3 Apparatus

Auditory stimuli were delivered using Sennheiser MX 475 intra-aural earphones, which fit in the concha at the entrance to the external auditory canal. EEG recordings were carried out using 64-channel Active Two system (BioSemi, Amsterdam, NL), with a 10–20 system headcap and four additional leads located above and below the right eye and in the external canthi of both eyes. Two additional reference electrodes were placed on mastoids and recorded in parallel. CMS and DRL electrodes were placed between POz and PO3 and POz and PO4 respectively. Data were sampled at 1024 Hz. Stimulus presentation was managed by Presentation software (Neurobehavioral Systems, Berkeley, USA).

### 2.4 EEG data processing

EEGLAB and ERPWAVELAB working in the MATLAB environment (Delorme and Makeig 2004; Mørup et al. 2007) were used for the off-line processing of EEG. The power-line noise was removed using multi-tapering and Thomas F-statistics implemented in CleanLine plugin for EEGLAB (https://www.nitrc.org/projects/cleanline). The outer range electrodes were rejected from further evaluation, remaining 41 electrodes ('AF3′, 'AFz', 'AF4′, 'F6′, 'F4′, 'F2′, 'Fz', 'F1′, 'F3′, 'F5′, 'FC5′, 'FC3′, 'FC1′, 'FCz', 'FC2′, 'FC4′, 'FC6′, 'C6′, 'C4′, 'C2′, 'Cz', 'C1′, 'C3′, 'C5′, 'CP5′, 'CP3′, 'CP1′, 'CPz', 'CP2′, 'CP4′, 'CP6′, 'P6′, 'P4′, 'P2′, 'Pz', 'P1′, 'P3′, 'P5′, 'PO3′, 'POz', 'PO4′). Channels with excessive noise were determined by visual inspection and rejected. On average 36 (± 3 SD) channels remained after rejection. Removed channels were replaced using spherical spline interpolation of the voltage from surrounding electrodes (Perrin et al. 1989). Correction of eye-movements was performed using Independent Component analysis (ICA), as implemented in EEGLAB (with default settings). Data were resampled to 512 Hz and re-referenced to an average reference. The epochs of 1500 ms were created starting at 500 ms prior to the stimulus onset and lasting for 1000 ms post-stimulus onset. The signal contained with the time-window of ASSR response was baseline-corrected to the mean of the pre-stimulus period.

In the next step, a wavelet transformation (WT) with complex Morlet wavelet (with 7 cycles) routine from Matlab Wavelet Toolbox was performed with signal frequencies represented from 1 to 120 Hz, and with 1 Hz intervals between each frequency. Inter-trial phase coherence (phase-locking index, PLI) was computed (Mørup et al. 2007) using ERPWAVELAB. The relative baseline correction for PLI was carried out by dividing the signal by baseline activity (averaged from −400 to −100 ms); in this way 1 means that PLI of the baseline is equal to PLI during stimulation.

Only data from FCz electrode were selected for further processing. This decision was based on our previous work in healthy volunteers (Pipinis et al. 2018) with the same type of stimuli, as well as on many other ASSR studies that consistently demonstrate that the strongest ASSR response can be detected at the frontocentral location on the scalp (Plourde & Picton, 1990, Ross et al. 2003, Dimitrijevic & Ross 2008, Binder et al. 2017).

### 2.5 Data analysis

The diagonal representing the time-frequency points of the stimulation was used to define the exact time point for each stimulation frequency. Starting from these points, EEG responses to the sound frequencies (from 120 to 1 Hz in 1 Hz step) were calculated as the average in the time windows covering the sound onset time for a given frequency and terminating after 100 ms delay (thus covering the response time based on the observation of grand-averaged TF plots) resulting in PLI-frequency curves.

In order to perform correlational analyses between PLI values and CRS-R scores while effectively controlling the type I error in a situation involving multiple comparisons we used a non-parametric cluster-based permutation procedure, implemented in FieldTrip software (Oostenveld et al. 2011). Pearson correlation was calculated for all CRS-R subscales and total scores and PLI values, separately. Samples that survived the initial test (i.e. the uncorrected p-value was less than 0.005) were clustered based on the spectral proximity. Cluster-level statistics were obtained by summing the frequency sample statistics within each cluster. The maximum of these was used to test the significance of our results against a randomization distribution. This distribution was obtained by randomly permuting the original data, taking the maximum cluster-level statistic and repeating this process for 20,000 times. The probability of obtaining a statistic from this distribution larger than the actual cluster statistic was tested at p level set less than 0.05.

## 3 Results

**Fig. 2:**
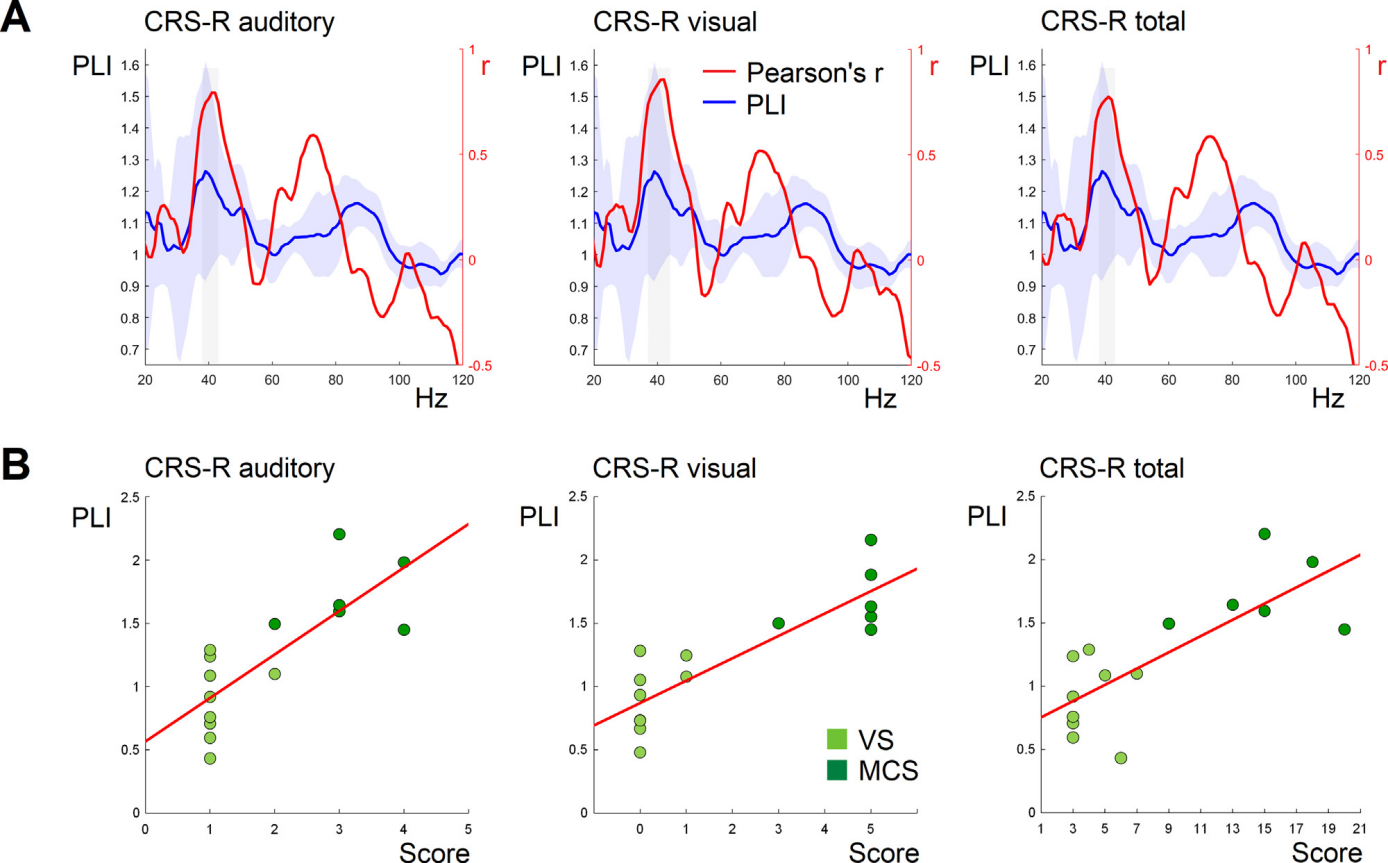
A: The course of the Pearson's correlation coefficient r (red line) between the PLI of the response and the auditory, visual and total CRS-R scores. The gray area highlights the frequency window within which the significant correlation between PLIs and CRS-R scores was indicated by the cluster-based permutation test. The light blue ribbon around the average PLI curve indicates the standard deviation. **B:** The scatterplots of PLI values averaged in the significant windows (as highlighted above) against individual scores.

**Fig. 3:**
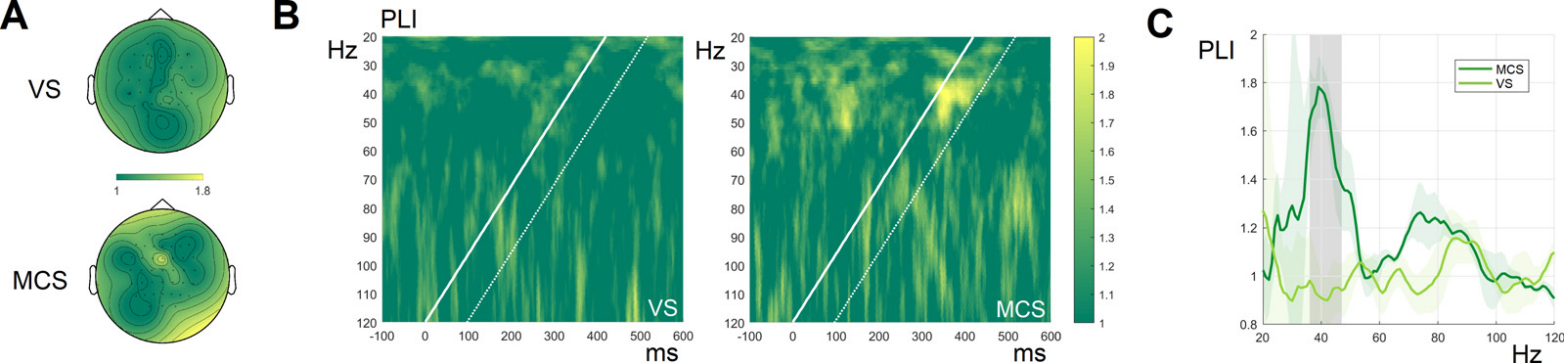
A: Topographies of grand-averaged PLIs in the 36–47 Hz frequency window separately for VS and MCS subgroups. **B:** Time-frequency plot of PLIs in VS (*N* = 9) and MCS (*N* = 6) groups. The white solid line corresponds to the stimulation, the white dashed line depicts +100 ms window. **C:** The PLI grand-averaged curves drawn separately for MCS and VS groups. gray area denotes a period of significant difference between PLIs of MCS and VS groups. The light green ribbons around the average PLI curve in each group indicate the standard deviation.

As shown by non-parametric cluster-based permutation procedure, the significant correlations between PLI estimates and CRS-R scores were found within a narrow 37–43 Hz window. The significant positive association was discovered for Auditory (38–43 Hz range, *p* = 0.016 and 0.69 < *r* < 0.79), Visual (37–43 Hz range, *p* = 0.008, 0.70 < *r* < 0.85) and total CRS-R score (38–42 Hz, *p* = 0.020, 0.71 < *r* < 0.76). The PLI curve and the course of corresponding r values for Auditory, Visual and Total CRS-R scores are plotted in Fig. 2A. Based on the scatterplot of PLI values against scores of the CRS-R results (Fig. 2B), the two subgroups could be distinguished, corresponding to the diagnoses of the patients - MCS and VS respectively. Grand averaged time-frequency plots of PLIs were created separately for these subgroups (Fig. 3C). We performed point-to-point comparison of PLI curves from MCS and VS subgroups. The non-parametric cluster-based permutation procedure revealed a significant difference between PLI values of these groups in the 36–47 Hz window (*p* = 0.017).

The scalp topography plots for the MCS group demonstrate (Fig. 3A) that the most prominent response in the low gamma range was observed in the frontocentral region, confirming validity of choosing FCz channel as the basis for estimating the effects of chirp-based auditory stimulation.

As indicated in Fig. 3B, the MCS group clearly showed the activity at the low gamma range (30–50 Hz) in response to chirp stimulation. On the contrary, VS group did not show chirp-evoked responses. This difference can also be seen in the comparison of grand averaged PLI curves depicted on Fig. 3C.

## 4 Discussion

This study aimed at the evaluation of the ability of injured brain networks to respond to low (30–50 Hz) and high (80–110 Hz) gamma frequencies, in a group of patients diagnosed with prolonged disorders of consciousness. Chirp-modulated tones were used to evoke responses and we expected that 1) in agreement with the previous study, phase-locking of in the low gamma (30–50 Hz) activity would correlate to the state of the patients as measured with CRS-R diagnostic tool, and 2) the response within low gamma range would demonstrate potential for the separation between vegetative and minimally conscious patients.

The phase-locking in the low gamma (37–43 Hz) window demonstrated strong correlation with the state of the patients as measured with the CRS-R total score, as well as the scores of CRS-R Auditory and Visual Function subscales. A significant difference in the PLIs was found between MCS and VS patients in the 36–47 Hz window, suggesting the potential of the chirp-evoked responses to separate these groups. We did not observe any differences between patient groups in the high gamma response within 80–110 Hz window and found no significant correlations with CRS-R scores.

The result of the present study replicates the main outcome of the previous report by our group (Binder et al. 2017) with the same sample, but with a different ASSR protocol involving 40 Hz click stimulation. In our experiment we have observed a correlation between PLIs of 40 Hz ASSRs and CRS-R total scores. The present chirp-based protocol supports sensitivity of the low gamma ASSR specifically to the state of a DOC patient, and thus encourages its use as an objective extension of diagnostic tools available to assess the state of the patients. This finding also goes in line with the earlier observations on the potential of 40 Hz ASSRs to index the severity of brain damage (Spydell et al. 1985, Harada et al. 1994), as well as the recent study by Chen et al. (2020) on using ASSR to predict prognosis for comatose patients. It has been also shown that 40 Hz ASSR is sensitive to changes in the level of arousal that occur naturally, i.e. during sleep (Górska & Binder, 2019; Tlumak et al. 2012, Cohen et al. 1991, Picton et al. 2003), or are artificially modulated with general anaesthetics (Plourde & Picton 1990, Plourde et al. 1998, for review see Plourde, 2006). This effect, though not shown comprehensively before, is supported by the observed decrease of the 40 Hz component in response to chirp stimulation in two participants during sleep (Artieda et al. 2004).

In the previous study we have proposed that the sensitivity of 40 Hz ASSR to patient state could be dependent on either attentional modulation of 40 Hz ASSR, or on the disruption of the intrinsic connectivity of the thalamo-cortical networks underlying entrainment of oscillations in the low gamma range. The studies using chirp stimulation have repeatedly demonstrated lack of sensitivity of phase-locking at the low gamma range to selective attention (Pipinis et al. 2018; Alegre et al. 2008). This strengthens the hypothesis that the susceptibility of the response at ∼40 Hz to the level of consciousness of the patients is not related to the attentional influence, but to a more fundamental aspect of brain function, namely, the functional integrity of thalamocortical networks. This conjecture is supported by the findings from naturally occurring states of changed consciousness level such as NREM sleep resulting in the decreased phase consistency of 40 Hz ASSR (Picton et al., 2003b, Lustenberger et al. 2018, Binder & Górska 2019). Potential mechanism of this decrease might involve tonic hyperpolarization of thalamocortical networks (McCormick & Bal 1997, Timofeev et al. 2001). The similarity between physiological states of low arousal and patterns of thalamocortical reactivity in patients with severe brain injury has been recently supported by Rosanova et al. (2018), who used TMS/EEG perturbation method in a group of VS/UWS patients. The transient TMS-evoked response waveforms revealed stereotyped, non-complex waveforms and prolonged OFF periods with decreased neuronal firing and suppressed spontaneous oscillations above 20 Hz, indicating lowered neural signal propagation capacity and a limited potential to generate complex activity. The pattern was similar to that in the states of lowered arousal, such as non-REM sleep, that are known to be caused by extensive tonic hyperpolarization of thalamocortical networks. It is then possible that lower PLI values of the low gamma in VS/UWS subgroup might be caused by a shift of inhibition/excitation balance in thalamocortical networks towards inhibition; this in turn may result from the injured dorsal brainstem sources or from an extensive damage to thalamocortical networks, resulting in reduced influence of ascending activation systems (Meythaler et al. 2001, Rosanova et al. 2018).

Also in line with the previous study, a significant correlation between PLIs of the low gamma responses to Auditory and Visual Function subscales was observed. These subscales require an efficient speech comprehension to obtain better diagnosis using CRS-R (Bruno et al. 2012). Thus, low gamma response can potentially reflect the ability to understand verbal communication for which integrity of cortical networks projecting to and from auditory cortices is necessary (Alaerts et al. 2009, Dimitrijevic et al. 2004, Manju et al. 2014). As demonstrated by Braiman et al. (2018), EEG correlates of auditory processing integrity to natural speech envelope show a strong association with the behavioral diagnosis based on CRS-R, and may even predict ability to perform complex tasks based on auditory comprehension, such as the command following protocol in fMRI (Owen et al., 2006).

The high gamma-band (80–110 Hz) response did not correlate with the state of patients, and - as can be seen in Fig. 3– its presence is barely visible on averaged responses. The lack of observed response in the high gamma range could have been caused by the contribution from overlapping EMG activity in the patient group and thus decreased signal to noise ratio in the high gamma range.

Several limitations of the current study should be noted. The first is related to the relatively small sample of the patients. However, the strength of the observed correlations allows us to speculate that the investigation of a larger sample would confirm these initial results. Another issue is related to the behavioral assessment with CRS-R that was not administered on the day of the EEG measurement: it might possibly influence the strength of association between behavioral state of the patient and the function of the brain networks responsible for the low gamma response. However, the influence of this confounding factor is diminished by high test-retest reliability of CRS-R (Seel et al. 2010, Porta et al. 2013) and low score variability across several days (Schiff et al. 2007, Beukema et al., 2016).

## 5 Conclusions

A strong positive correlation between phase-locking in the low gamma range to chirp-modulated tones and the total CRS-R score in a group of prolonged DOC patients was observed together with a significant difference of PLI values between patients’ subgroups. Our results support the notion for the activity around 40 Hz to serve as a possible marker of the integrity of thalamocortical networks in prolonged DOC patients. The very narrow window of the activity paralleling CRS-R scores and repeatability of the observation with different assessment methods suggest a potentially high specificity of the proposed marker. Further studies in larger patient samples are needed to confirm these initial results.

## Acknowledgments

The study was supported by the Polish National Science centre under awards number UMO-2013/11/B/HS6/01,242, and UMO-2016/20/T/HS6/00,233.

## CRediT authorship contribution statement

**Marek Binder:** Conceptualization, Methodology, Software, Investigation, Resources, Data curation, Writing - original draft, Writing - review & editing, Supervision, Project administration, Funding acquisition. **Urszula Górska:** Investigation, Methodology, Data curation, Writing - review & editing. **Evaldas Pipinis:** Software, Formal analysis, Writing - original draft. **Aleksandras Voicikas:** Software, Formal analysis. **Inga Griskova-Bulanova:** Methodology, Formal analysis, Writing - original draft.
